# Toxicity of gold nanoparticles complicated by the co-existence multiscale plastics

**DOI:** 10.3389/fmicb.2024.1447046

**Published:** 2024-08-29

**Authors:** Lan Zhang, Yuyang Ma, Zhiliang Wei, Luyang Wang

**Affiliations:** ^1^College of Food Science and Engineering, Ocean University of China, Qingdao, China; ^2^School of Pharmacy, Binzhou Medical University, Yantai, China; ^3^Department of Radiology & Radiological Science, Johns Hopkins University School of Medicine, Maryland, MD, United States

**Keywords:** gold nanoparticles, microplastics, nanoplastics, toxicity, gut microbiota

## Abstract

**Introduction:**

Gold nanoparticles (AuNPs) have been developed as treatment materials for various diseases and shown magnificent potential. By contrast to the broad toxicological studies on the single exposure (AuNPs), how the other health risks modulate the toxicological profile of AuNPs remains to be investigated. Plastics are among the most common health risks in daily life due to the broad utilization of plastic products. Therefore, in this study, we aimed to reveal the toxicological effects induced by co-exposure of gold nanorod (AuR) and polystyrene micro- and nano-plastics (hereinafter, referred to as AuRmPS and AuRnPS, respectively) in mice.

**Methods:**

Systematic biochemical characterizations were performed to investigate the hepatotoxicity, nephrotoxicity, neurotoxicity, inflammatory responses, alterations in gut microbiota induced by co-exposure, and to analyze the toxicological phenomena from the roles of reactive oxygen species and gut-organ axis.

**Results:**

It has been found that hepatotoxicity, nephrotoxicity, neurotoxicity, and inflammation were exacerbated in AuRnPS and AuRmPS, and gut microbiota composition was more severely altered in AuRnPS exposure. These results suggest the necessity of reducing plastics exposure in AuNPs-based therapies. Moreover, protection against the nano-sized plastic particles holds higher priority.

**Conclusion:**

These findings will facilitate the explorations of methods to reduce therapeutic toxicity and improve biosafety for specific treatments by referring to the orders of importance in protecting different organs.

## 1 Introduction

**Figure 1:**
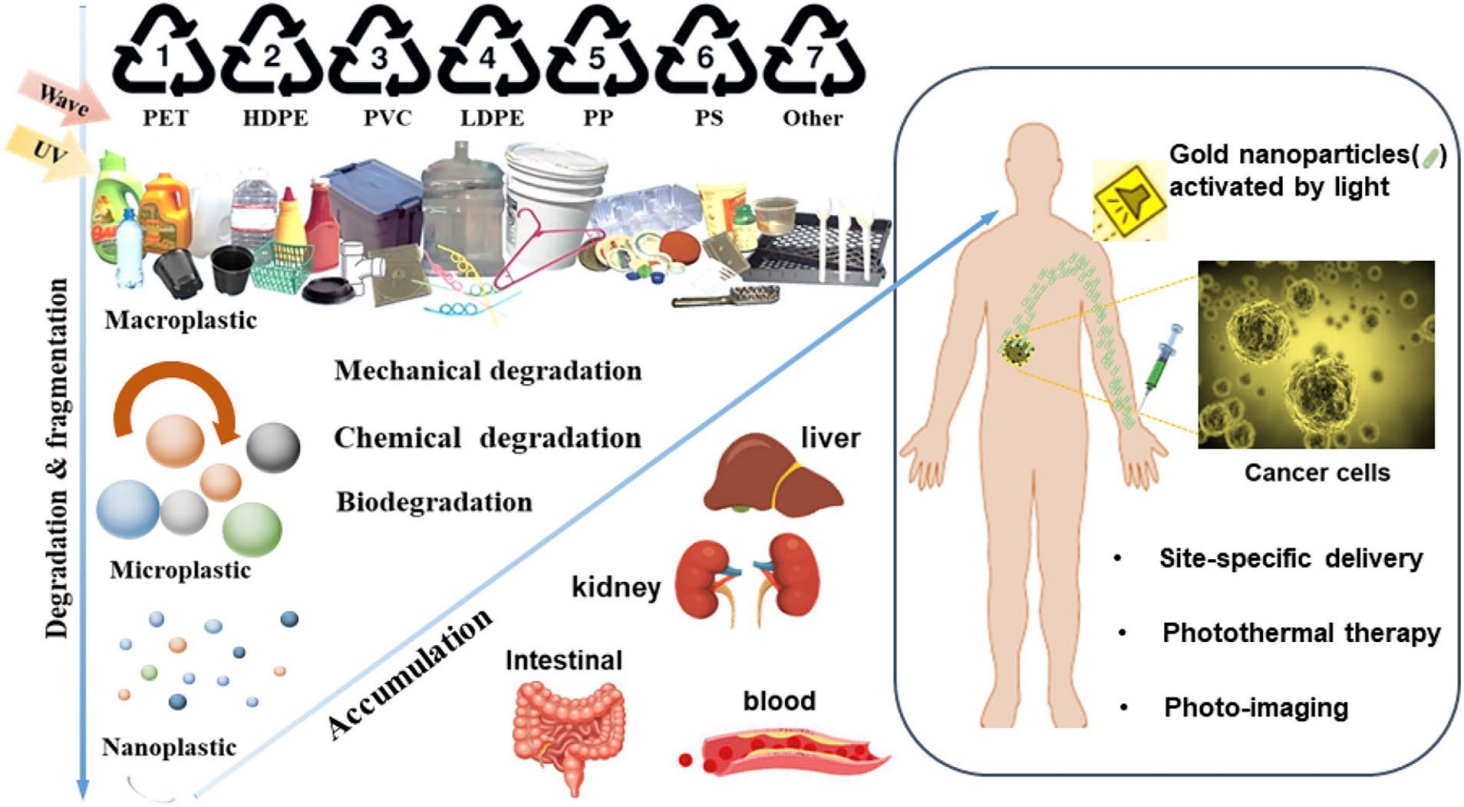
Schematic illustration for the fates of micro−/nanoplastics and gold nanoparticles (AuNPs). Macroplastic are fragmented and degraded into micro−/nano-plastic due to various mechanical, chemical and biological processes in the environment. Micro−/nano-plastics can enter the human bodies and coexist with AuNPs.

Triggered by the discovery of immunogold labeling, the biomedical applications of metal nanoparticles are attracting more and more interests since 1970 (Faulk and Taylor, 1971). Among these applications (Requejo et al., 2019; Zhang et al., 2019), gold nanoparticles (AuNPs) have been involved in the photo-diagnostics and photothermal therapy (Huff et al., 2007; An et al., 2017; Figure 1). AuNPs are associated with excellent surface affinity allowing for facile bioconjugation with a variety of biomolecules to promote drug delivery (Boisselier and Astruc, 2009; Figure 1). Due to its unique property in plasmon resonance, AuNPs have been developed to be a remarkable imaging label and contrast agent to improve the diagnostics of cancer (Eghtedari et al., 2007), Alzheimer’s disease (Haes et al., 2005), and HIV (Mahmoud and Luong, 2008) etc. Apart from the applications as diagnostic tools, there are surging evidences demonstrating the versatility of AuNPs in treatments, including photothermal cancer therapy (Brigger et al., 2002; Jung et al., 2013), angiogenesis therapy (Bhattacharya and Mukherjee, 2008), anti-bacterial therapy (Zharov et al., 2006), and drug vectorization (Paciotti et al., 2004; Visaria et al., 2006; Han et al., 2007) etc. AuNPs are playing a role with increasing importance based on its potentials in diagnoses and treatments, and human exposure to AuNPs is becoming more prevalent. Accordingly, toxicological studies should be performed to investigate the biosafety of AuNPs in more depth and width.

Plastics usage is a worldwide health risk as revealed by the findings of micro- and nano-plastics in water, soil, organisms, and daily diet (primarily through the food chain; Eriksen et al., 2014; Geyer et al., 2017; Figure 1). Daily usage of plastics products is a major contributor to the accumulation of plastics particles. Due to the COVID-19 pandemic, the plastic use has increased globally, including the inevitable utilization of disposable protection suite and additional covers to prevent virus infections (Ray et al., 2022). Polystyrene (PS) is one of the most widely used plastics for food industrial packaging, cosmetics, and medical applications (Fendall and Sewell, 2009; Banerjee and Shelver, 2021; Zarus et al., 2021). PS particles has been reported to induce multifaceted responses, e.g., dysbiosis, inflammation, and growth inhibition etc. (Lu et al., 2018; Li et al., 2020; Teng et al., 2022). Therefore, the potential influence of micro−/nanoscale PS particles in therapies is worthy of attention.

Toxicity of single exposure (i.e., AuNPs) has been well studied in terms of shapes (Chithrani et al., 2006), sizes (Chithrani et al., 2006; Chen et al., 2009), and chelate structures (Connor et al., 2005; Taylor et al., 2012) etc. However, there is a lack of investigations focusing on co-exposures with other health risk factors. Toxicological study on the co-exposure of AuNPs and PS will convey special importance for AuNPs-based therapies or therapeutic monitoring where repeated exposures are inevitable. Some studies have reported that PS nanoplastics induced only marginal effects on the survival, hatching rate, developmental abnormalities, and cell death of zebrafish embryos but that these effects were synergistically exacerbated by the Au ion in a dose- and size-dependent manner (Lee et al., 2019). Such exacerbation of toxicity was well correlated with the production of reactive oxygen species and the proinflammatory responses synergized by the presence of PS, supporting the combined toxicity of PS and Au ions. The synergistic effect of PS on toxicity appeared to relate to mitochondrial damage as determined by ultrastructural analysis (Lee et al., 2019). Taken together, the effects of PS nanoplastics were marginal but could trigger for exacerbating the toxicity induced by other toxicants such metal ions (Lee et al., 2019). Fu et al. found that microplastic-contaminated antibiotics as an emerging threat enhanced oxidative and inflammatory damages to livers (Fu et al., 2023). In another study, microplastics aggravate the toxicity of organophosphorous flame retardants in mice (Deng et al., 2018). Moreover, conventional toxicological studies primarily focused on regular biochemical properties solely and there is a lack of observation of these biochemical properties in the context of gut-organ axes, which are associated with critical roles in various pathogenesis.

Therefore, in this study, we aim to fill these gaps by investigating the toxicological profile of co-exposure between AuNPs and PS in unison with single AuNPs exposure. Conventional biochemical markers were compared in the context of gut microbial alterations to answer some basic but critical questions in AuNPs-based therapies: Is it necessary to control plastics exposure in AuNPs-based therapies? Is there a dependence of toxicity on the PS particle size? We investigated the exposure of gold nanorod (AuR) with two commercial PS particles in different sizes (500 nm and 5 μm) in mice. Systematic biochemical characterizations were performed to investigate the hepatotoxicity, nephrotoxicity, neurotoxicity, inflammatory responses, lipid metabolism, and alterations in gut microbiota. By systematically evaluating the toxicity of gold nanoparticles and micro−/nano-plastic that are closely related to people’s daily life and medical treatment, this study helps us to minimize the harm while exerting the beneficial side of nanomaterials to contribute to mankind. This study has important reference value for the biotoxicity of nanoparticles *in vivo* and has important guiding significance for their application in the field of biomedicine.

## 2 Materials and methods

### 2.1 Apparatus and chemicals

Extinction spectra was collected with a UV-1800 spectrophotometer (Shimadzu). Transmission and scanning electron microscopes (TEM and SEM) were used. Biochemical markers were measured with Varioskan Flash (Thermo Scientific). Gold (III) chloride trihydrate and cetyltrimethylammonium bromide (CTAB; Sigma-Aldrich, Burlington, United States), ascorbic acid, silver nitrate, and sodium borohydride (Sinopharm Chemical Reagent, Ningbo, CHN), micro−/nano-polystyrene plastics (5 μm and 500 nm; Macklin Biochemical, Shanghai, CHN), and commercial kits (Jiancheng Bioeng. Inst., Nanjing and Calvin Biotechnology, Suzhou, CHN) were purchased and used.

### 2.2 Experimental design

AuR with dimensions of 50 × 20 nm (length × diameter) was prepared using El-Sayed’s seed growth method (Nikoobakht and El-Sayed, 2003) and used at a concentration of 30 mg/L. To consider the size effect on biological toxicity, plastic particles in the sizes of 500 nm and 5 μm were utilized to represent the nanoscale and microscale plastic pollutants (Liu et al., 2021; Jeong et al., 2022), respectively. PS of different sizes (500 nm, denoted as nPS, 5 μm, denoted as mPS) were diluted with ultrapure water and mixed with AuR to reach a concentration of 30 mg/L. Dosage was determined based on previous studies (Besseling et al., 2014; Li et al., 2019; Cheng et al., 2022; Fu et al., 2023).

### 2.3 Animals and sample collection

The experimental protocol was approved by the Institutional Animal Care and Use Committee of the Laboratory Animal Ethics Review Form of the College of Food Science and Engineering, Qingdao Ocean University, China (approval number: SPXY2020101301). The experimental animal kept site and experimental site were carried out in the animal room on the 62nd floor of the Yushan Campus of Ocean University of China and the nucleic acid chemistry and biotechnology laboratory in the Minxing Hall, respectively. A total number of 32 SPF (Specific Pathogen Free) grade ICR (Institute for Cooperative Research) mice (5 weeks old) were purchased from Jinan Pengyue Laboratory Animal Breeding Co., Ltd. The mice were acclimated for 1 week prior to the study and cultured in a standard 12 h-light/12 h-dark cycle at 24 ± 4°C in a relative humidity of 60% ± 4% throughout the experiments. Mice were randomly divided into four groups (*N* = 8 for each group with balanced gender): (1) Con group, i.e., control group receiving normal water feeding; (2) AuR group receiving AuR; (3) AuRnPS group receiving AuR and 500 nm PS; (4) AuRmPS group receiving AuR and 5 μm PS. Solutions were fed by oral gavage at a dose of 10 mL/kg body weight for 90 consecutive days with weekly recording of body weight.

Two days before the autopsy, feces were collected and stored at −80°C for gut microbiota analysis. After the 90-day exposure, mice were euthanized for organ (heart, liver, spleen, lung, kidney, and brain) and blood collections. Tissue samples were rinsed with 0.9% saline to remove blood stains, dried with filter paper, and weighted with a balance. Fractions of liver and kidney were stored for histopathological analyses. The remaining liver tissues and brain were pretreated with liquid nitrogen and stored at −80°C for analyses.

### 2.4 Histopathological analysis

The mouse liver and kidney tissues obtained from four groups were fixed in 4% formalin and embedded in paraffin wax and frozen liver and kidney tissue section were prepared as described previously (Wang et al., 2021). Each of the tissue samples were sectioned at a thickness of 7 μm, and then stained with hematoxylin and eosin (H&E) staining kit for microscopic observation according to the manufacturer’s instructions. The staining procedure for H&E follows a basic protocol: Dewaxing; Dehydration; Hematoxylin; Differentiation; Bluing; Eosin; Dehydration; Clearing; and Cover-slipping.

### 2.5 Biochemical analyses

For each of the liver samples, superoxide dismutase (SOD), catalase (CAT), glutathione (GSH), and malonaldehyde (MDA) were determined by using commercial kits to investigate hepatotoxicity. Activities of alanine aminotransferase (ALT) and aspartate amino-transferase (AST) were measured with blood serum by using commercial kits to gain insight into liver damage. For each of the kidney samples, the contents of creatinine (CRE) and blood urea nitrogen (BUN) were measured by using commercial kits to investigate nephrotoxicity. Acetylcholinesterase (AChE) activity and nitric oxide (NO) content of mice brains were detected by using commercial kits. Triglyceride (TG), the total cholesterol (T-CHO), high-density lipoprotein cholesterol (HDL-C), and low-density lipoprotein cholesterol (LDL-C) were measured with blood serum to gain insight into lipid metabolism. Commercial biochemical kits (Nanjing Jianchen, Suzhou, CHN) were used to detect these biomarkers according to the manufacturer’s instructions.

Levels of tumor necrosis factor (TNF-α) and Interleukin-6 (IL-6) of the mice serum were determined by using double antibody one-step sandwich enzyme-linked immunosorbent assay (ELISA) kits according to the manufacturer’s instructions (Calvin Biotecnology, Suzhou, CHN) to gain insight into inflammation. The measuring procedure for ELISA follows a basic protocol: (1) Prepare all reagents before starting assay procedure. (2) Add standard: set standard wells, testing sample wells. Add standard 50 μL to standard well. (3) Add sample: add testing sample 10 μL then add sample diluent 40 μL to testing sample well; blank well does not add anything. (4) Add 100 μL of HRP-conjugate reagent to each well, cover with an adhesive strip and incubate for 60 min at 37°C. (5) Aspirate each well and wash, repeating the process four times for a total of five washes. Wash by filling each well with Wash Solution (400 μL) using a squirt bottle, manifold dispenser or autowasher. Complete removal of liquid at each step is essential to good performance. After the last wash, remove any remaining Wash Solution by aspirating or decanting. Invert the plate and blot it against clean paper towels. (6) Add chromogen solution A 50 μL and chromogen solution B 50 μL to each well. Gently mix and incubate for 15 min at 37°C. Protect from light. (7) Add 50 μL Stop Solution to each well. The color in the wells should change from blue to yellow. If the color in the wells is green or the color change does not appear uniform, gently tap the plate to ensure thorough mixing. (8) Read the Optical Density (O.D.) at 450 nm using a microtiter plate reader within 15 min. The data were obtained by using a Varioskan Flash Reader (Thermo Fisher Scientific, United States).

### 2.6 Gut microbiota analysis

#### 2.6.1 DNA extraction and PCR amplification

Microbial community genomic DNA was extracted from mouse feces samples using the PF Mag-Bind Stool DNA Kit (Omega Bio-tek, Georgia, United States) according to manufacturer’s instructions. The DNA extract was checked on 1% agarose gel, and DNA concentration and purity were determined with NanoDrop 2000 UV–vis spectrophotometer (Thermo Scientific, Wilmington, United States). For bacterial community, the bacterial 16S rRNA genes were amplified using the universal bacterial primers 338F (5’-ACTCCTACGGGAGGCAGCAG-3′) and 806R (5’-GGACTACHVGGGTWTCTAAT-3′). Primers were tailed with PacBio barcode sequences to distinguish each sample. Extracted DNA samples were amplified from the V3-V4 region of 16S rRNA and sequenced by Majorbio Biotechnology (Shanghai, CHN). High throughput sequencing technology was conducted to reveal the alterations of gut microbiota. Amplification reactions (20-μL volume) consisted of 5 × FastPfu buffer 4 μL, 2.5 mM dNTPs 2 μL, forward primer (5 μM) 0.8 μL, reverse primer (5 μM) 0.8 μL, FastPfu DNA Polymerase 0.4 μL, template DNA 10 ng and DNase-free water. The PCR amplification was performed as follows: initial denaturation at 95°C for 3 min, followed by 27 cycles of denaturing at 95°C for 30 s, annealing at 60°C for 30 s and extension at 72°Cfor 45 s, and single extension at 72°C for 10 min, and end at 4°C (ABI GeneAmp^®^ 9,700 PCR thermocyclerm, CA, United States). PCR reactions were performed in triplicate. After electrophoresis, The PCR products were purified using the AMPure^®^ PB beads (Pacifc Biosciences, CA, United States) and quantified with Quantus^™^ Fluorometer (Promega, WI, United States).

#### 2.6.2 DNA library construction and sequencing

Purified products were pooled in equimolar and DNA library was constructed using the SMRTbell prep kit 3.0 (Pacifc Biosciences, CA, United States) according to PacBio’s instructions. Purified SMRTbell libraries were sequenced on the Pacbio Sequel IIe System (Pacifc Biosciences, CA, United States) by Majorbio Bio-Pharm Technology Co. Ltd. (Shanghai, China).

#### 2.6.3 Data processing

PacBio raw reads were processed using the SMRTLink analysis software (version 11.0) to obtain high-quality Hifi reads with a minimum of three full passes and 99% sequence accuracy. Hifi reads were barcode-identified and length-filtered. For bacterial 16S rRNA gene, sequences with a length < 1,000 or > 1,800 bp were removed. The Hifi reads were clustered into operational taxonomic units (OTUs) using UPARSE 11 (Stackebrandt and Goebel, 1994; Edgar, 2013) with 97% sequence similarity level. The most abundant sequence for each OTU was selected as a representative sequence. The OTU table was manually filtered, i.e., chloroplast sequences in all samples were removed. To minimize the effects of sequencing depth on alpha and beta diversity measure, the number of 16S rRNA gene sequences from each sample were rarefied to 6,000, which still yielded an average Good’s coverage of 99.09%, respectively. The taxonomy of each OTU representative sequence was analyzed by RDP Classifier version 2.13 (Wang Q. et al., 2007) against the 16S rRNA gene database using confidence threshold of 0.7.

The metagenomic function was predicted by PICRUSt2 (Phylogenetic Investigation of Communities by Reconstruction of Unobserved States; Douglas et al., 2020) based on OTU representative sequences. PICRUSt2 is a software containing a series of tools as follows: HMMER was used to aligns OTU representative sequences with reference sequences. EPA-NG and Gappa were used to put OTU representative sequences into a reference tree. The castor was used to normalize the 16S gene copies. MinPath was used to predict gene family profiles and locate into the gene pathways. Entire analysis process was accord to protocols of PICRUSt2. Functional prediction of 16S amplicon sequencing results was performed by PICRUSt2 software package. Firstly, the OTU abundance table was standardized by PICRUSt (the PICRUSt process stored the COG information and KO information corresponding to the greengene id), that is, the effect of the number of copies of the 16S marker gene in the species genome was removed. Then, the KEGG Ortholog (KO) information corresponding to the OTU was obtained through the greengene id corresponding to each OTU. The abundance of each KO was calculated. The KEGG database (Kyoto Encyclopedia of Genes and Genomes)^1^ is a large repository of knowledge for the systematic analysis of gene function, linking genomic information and functional information. Based on the information in the KEGG database, KO and Pathway information can be obtained, and the abundance of each functional class can be calculated based on the OTU abundance. For the pathway, PICRUSt can be used to obtain the information of three levels of metabolic pathways, and the abundance table of each level can be obtained separately. The KEGG Pathway database includes various metabolic pathways, synthetic pathways, membrane transport, signaling, cell cycle, and disease-related pathways.

Software: PICRUSt2.^2^

### 2.7 Statistical analysis

All data were shown as mean ± standard error. Statistical analysis was performed using one-way ANOVA and Tukey’s test with Prism 8.0.2. A *p*-value <0.05 was considered as significant. Bioinformatic analysis of the gut microbiota was carried out using the Majorbio Cloud platform.^3^ Based on the OTUs information, rarefaction curves and alpha diversity indices including observed OTUs, Chao1 richness, Shannon index, Simpson index, Chao index, and Ace index were calculated with Mothur v1.30.2 (Schloss et al., 2009). The similarity among the microbial communities in different samples was determined by principal coordinate analysis (PCoA) based on Bray–curtis dissimilarity using Vegan v2.4.3 package. The correlation between microbial taxa and biochemical biomarkers was performed in the environmental factor association analysis module of Majorbio Biotechnology (Shanghai, CHN) data processing platform (software: R, version 3.3.1; pheatmap package).

## 3 Results

### 3.1 Characterizations of AuR and PS particles

**Figure 2:**
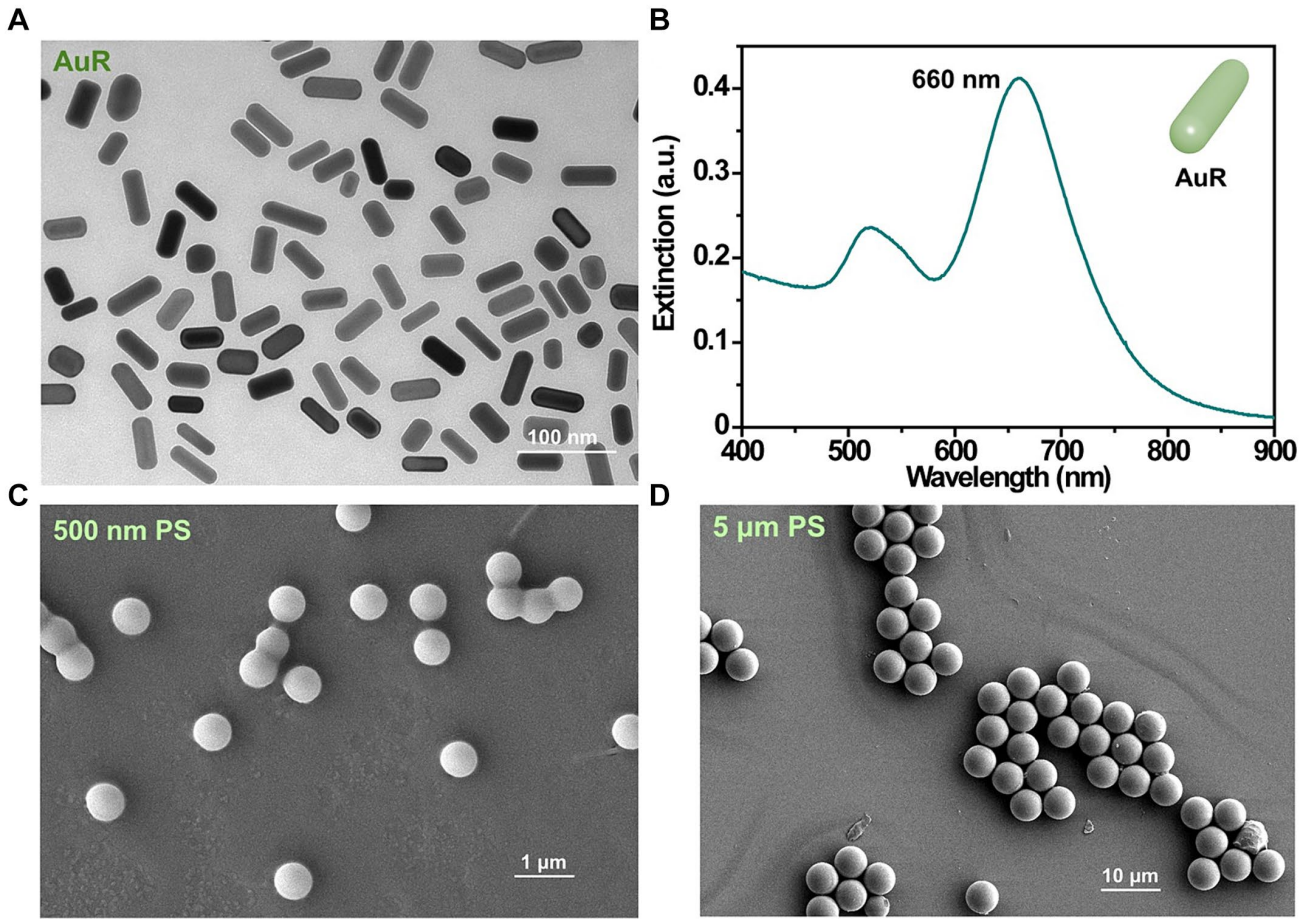
Characterizations of the AuR and PS used in this study. TEM image **(A)** and extinction spectra **(B)** of AuR. SEM images of 500 nm PS **(C)** and 5 μm PS **(D)**.

AuR exhibited a dimension of approximately 50 nm × 20 nm (length × diameter; Figure 2A) and a plasma peak at 660 nm (Figure 2B). The nPS and mPS were featured by diameters of about 500 nm (Figure 2C) and 5 μm (Figure 2D), respectively.

### 3.2 Hepatotoxicity, nephrotoxicity, and neurotoxicity profiles

**Figure 3:**
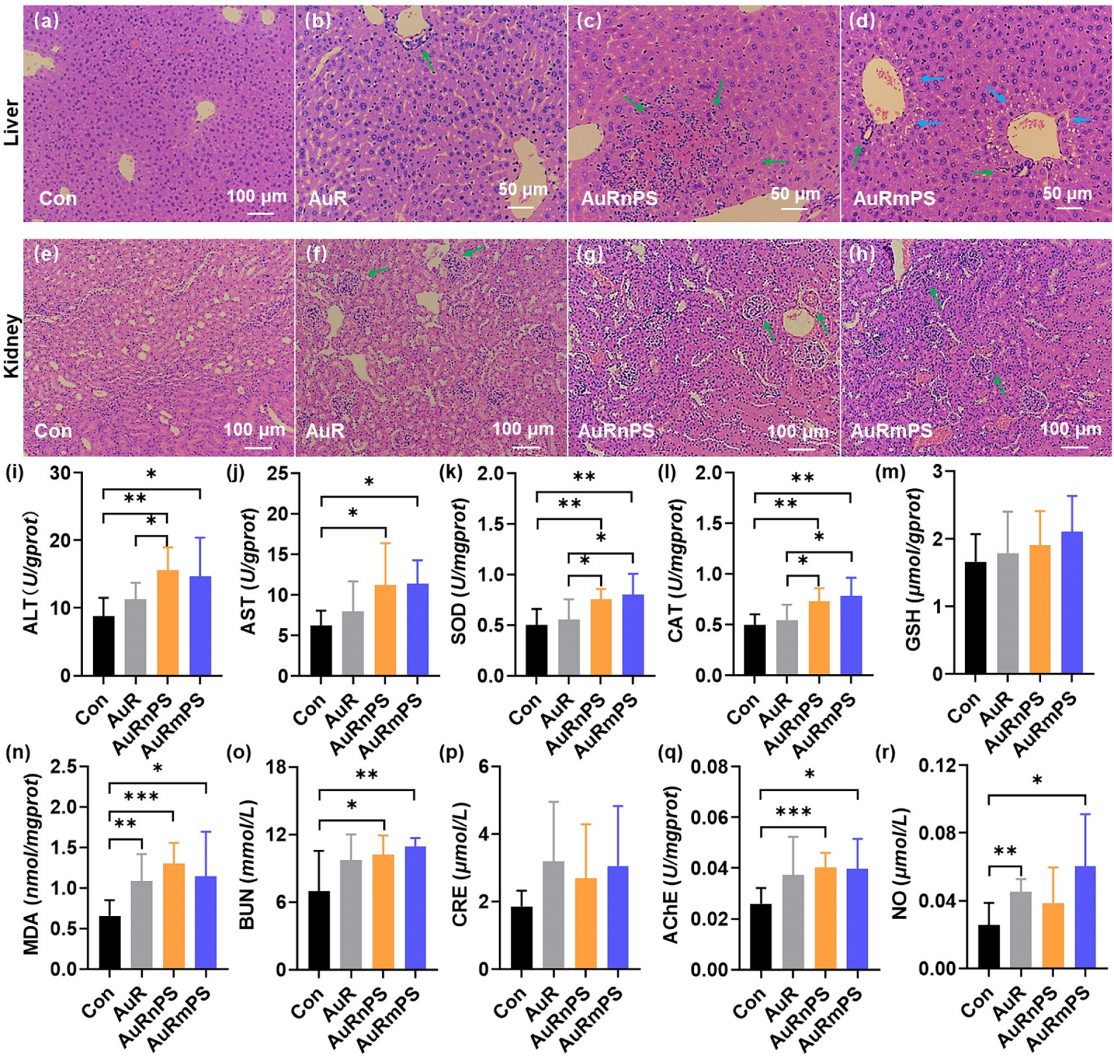
Histopathological results of liver **(A–D)**, and of kidney **(E–H)** for the Con, AuR, AuRnPS, and AuRmPS groups. Effects of single exposure (AuR) and co-exposures (AuRnPS and AuRmPS) on hepatotoxicity **(I–M)**, nephrotoxicity **(N,O)**, and neurotoxicity **(P,Q)**. **(I)** shows the comparisons among four groups for ALT; **(J)** for AST; **(K)** for SOD; **(L)** for GSH; **(M)** for CAT; **(N)** for MDA; **(O)** for BUN; **(P)** for CRE, **(Q)** for AChE, and for **(R)** NO. Green and blue arrows indicate inflammatory cell infiltration and fat vacuoles, respectively. *p < 0.05; **p < 0.01; ***p < 0.001.

H&E staining revealed significant changes in the liver and kidney cells upon single exposure and co-exposures (AuRnPS and AuRmPS). There were no noticeable morphometric abnormalities for the Con group (Figures 3A,E). Inflammatory cell infiltration (green arrows) was a common finding (green arrows in Figures 3B–D,F) in both liver and kidney. Fat vacuoles (blue arrows) were observed in the liver of AuRmPS mice (blue arrows in Figure 3D). The presences of liver and kidney damages were also supported by organ coefficients. Co-exposure groups exhibited significantly lower liver and kidney organ coefficients (*p* < 0.05) than the Con group (Supplementary Figure S1). There were no systematic organ coefficient alterations in other organs (Supplementary Figure S1), except for significantly decreased spleen coefficient in AuR mice and heart coefficient (*p* < 0.05) in AuRmPS mice (Supplementary Figure S1), indicating that liver and kidney were the most vulnerable organs to toxicity induced by co-exposures. In addition, body weights were similar in all groups (Supplementary Figure S1), suggesting that the organ damages were the outcome of toxicity instead of general body weight loss.

To further investigate the hepatotoxicity, the enzyme activities of transaminase, SOD, CAT, GSH, and MDA were measured. Co-exposure groups tended to possess abnormal transaminase activity, as shown by the significantly higher ALT (Figure 3I) and AST (Figure 3J) activities (*p* < 0.05) in comparison with the Con group. It can be inferred that the effect of AuR on transaminase activity has been enhanced by the co-exposure with PS. Co-exposure groups exhibited significantly higher SOD (Figure 3K) and CAT (Figure 3L) activities (*p* < 0.05) in comparison with the Con or AuR group. The GSH level was similar in all groups (Figure 3M). The toxic end-product of lipid peroxidation of reactive oxygen species (ROS), MDA was found to increase significantly in AuR, AuRnPS, and AuRmPS groups (*p* < 0.05, Figure 3N). These results demonstrated that exposure to AuR leads to altered oxidative stress profile and co-exposure with PS exacerbates the effect.

BUN and CRE, which are the end products of protein and creatine metabolism, were measured in blood serum to assess the glomerular filtration rate of kidney and provide a marker for renal function (Lewis et al., 2013). AuR group showed similar BUN and CRE contents compared with Con group (Figures 3O,P). By contrast, AuRnPS and AuRmPS groups were associated with significantly higher BUN content (*p* < 0.05, Figure 3O) but similar CRE content (Figure 3P) in comparison with Con group, suggesting that renal function was partially impaired by the co-exposure of AuR and PS.

Neurotoxicity in brain was assessed with the AChE activity and NO level. The AChE activity was found to increase significantly in AuRnPS and AuRmPS groups (*p* < 0.05) but not in AuR group (Figure 3Q), suggesting enhanced inhibitory responses to the neurotransmitter. There were NO level differences in AuR (*p* = 0.002) and AuRmPS groups (*p* = 0.011) but not in AuRnPS group (*p* = 0.16) in comparison with the Con group (Figure 3R), indicating that alterations in NO level depend on the PS size. These results support the neurotoxicity enhancement after co-exposure.

### 3.3 Inflammatory responses and lipid metabolism

**Figure 4:**
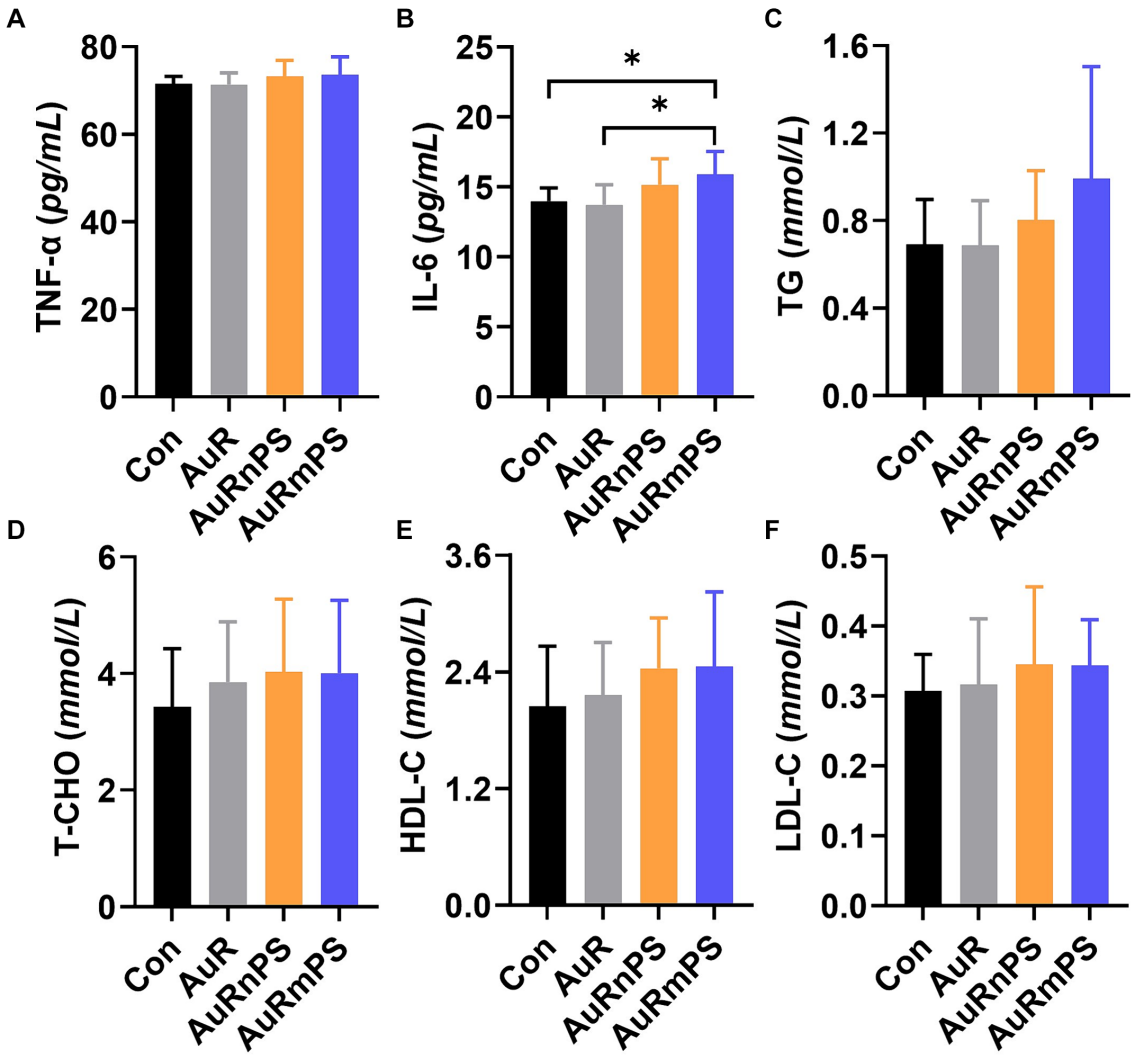
Effects of single exposure (AuR) and co-exposures (AuRnPS and AuRmPS) on inflammatory response **(A–C)** and lipid metabolism **(D–F)**. **(A)** shows the comparisons among four groups for TNF-α; **(B)** for IL-6; **(C)** for TG; **(D)** for T-CHO; **(E)** HDL-C, and **(F)** for HDL-C. *p < 0.05; **p < 0.01.

TNF-α levels were similar in all groups (Figure 4A). The AuRmPS group exhibited significantly (*p* < 0.05) higher IL-6 levels than the Con or AuR group (Figure 4B), possibly suggesting that inflammatory response is differentially modulated by co-exposures with different PS size. The levels of TG, T-CHO, HDL-C, LDL-C and the ratio between HDL-C and LDL-C content were similar in all groups (Figures 4C–F), suggesting that lipid metabolism is relatively intact.

### 3.4 Gut microbiota analyses

**Figure 5:**
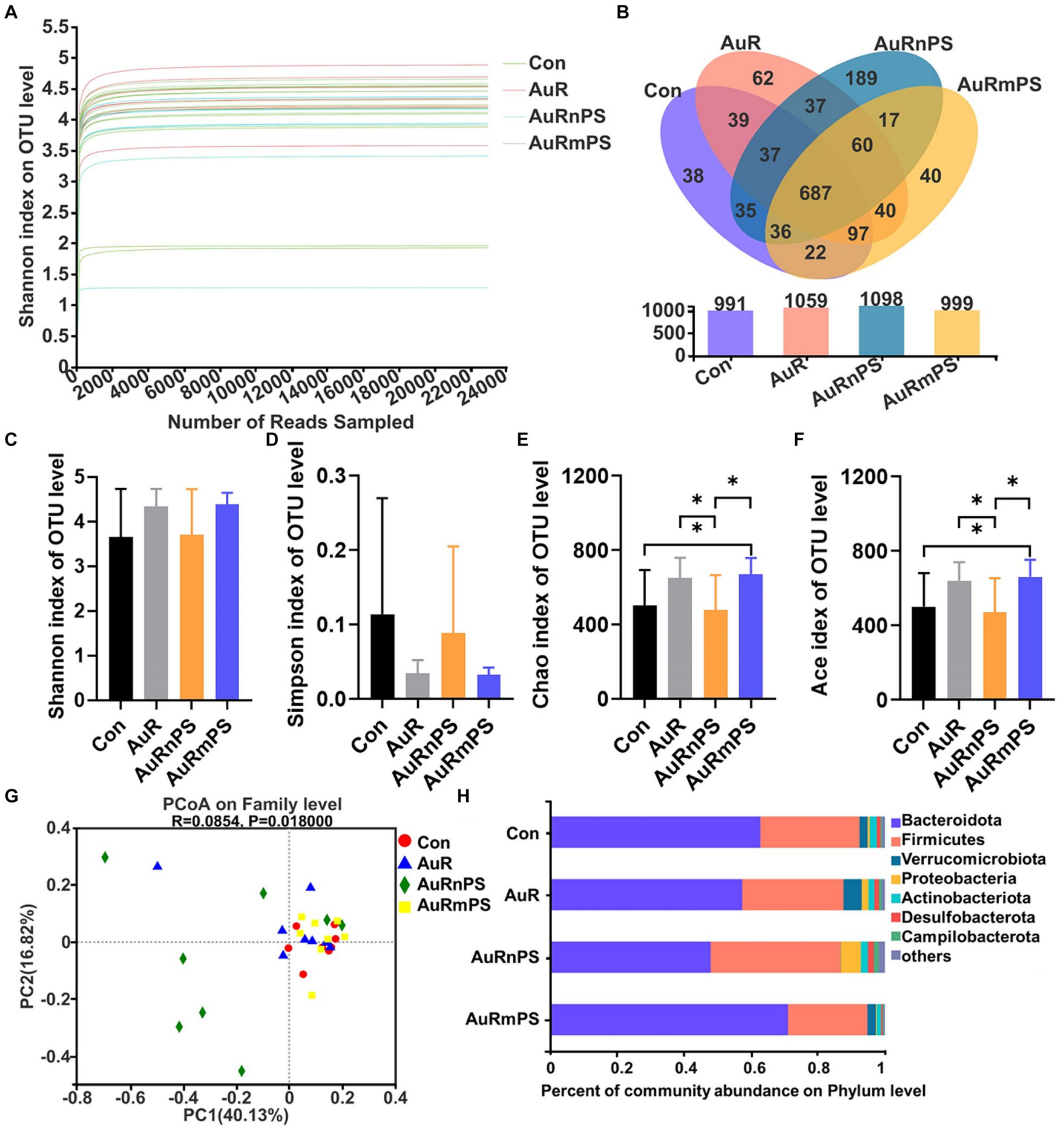
Effects of single exposure (AuR) and co-exposures (AuRmPS and AuRnPS) on community distribution and compositions of fecal microbiota. **(A)** Alpha index rarefaction curve. **(B)** Venn diagram for OTUs. **(C)** Shannon, **(D)** Simpson, **(E)** Chao, and **(F)** Ace indexes of OTU level; **(G)** Principal co-ordinates analysis (PCoA); and **(H)** Changes in the fecal microbiota composition at the family level. *p < 0.05.

A total number of 736, 480 high-quality 16S rRNA sequences were obtained from 32 samples, after subsampling each sample to an equal sequencing depth (23, 015 reads per sample) and clustering, 1,436 operational taxonomic units (OTUs) at 97% identity were obtained. According to the Shannon curves (Figure 5A), our sample reading number was sufficiently large to provide reliable evaluation on the microbial community components. Common and unique OTUs of four groups were summarized (Figure 5B). Total numbers of OTU in the Con, AuR, AuRnPS, and AuRmPS groups were 991, 1,059, 1,098, and 999, respectively. Shared OTU number by the four groups was 687, and AuRnPS group possessed the largest number of specific OTUs.

Similar Shannon and Simpson indexes (representing community diversity) were found in all groups (Figures 5C,D). The AuRmPS group exhibited significantly higher Chao (*p* = 0.038) and Ace indexes (*p* = 0.042; denoting community richness) in comparison with the Con group (Figures 5E,F). By contrast, the AuRnPS group showed significantly reduced Chao (*p* = 0.039) and Ace (*p* = 0.038) indexes in comparisons with the AuR or AuRmPS group (Figures 5E,F). The AuRnPS group can be well separated from the other groups (Con, AuR, and AuRmPS) with the PCoA model (Figure 5G). These results suggested that the perturbation on alpha and beta diversity of gut microbiota is related to the size of the co-exposed PS.

A column diagram was generated based on the relative abundances of species at the phylum level (Figure 5H). The top seven dominant bacteria with largest microbial abundances in mice were *Bacteroidota*, *Firmicutes*, *Verrucomicrobiota*, *Proteobacteria*, *Actinobacteriota*, *Desulfobacterota*, and *Campilobacterota* (Figure 5H). The top two leading microbiota (i.e., *Bacteroidota* and *Firmicutes*) accounted for ≥87% the total abundance for all groups. In comparison with the AuRmPS group, AuRnPS group was characterized by significantly lower *Bacteroidota* (*p* = 0.031) but higher *Firmicutes* (*p* = 0.044) and *Desulfobacterota* (*p* = 0.035; Supplementary Figure S2). Analyses at the family level showed a similar finding in that dominant microbial species was differentially altered in AuRnPS and AuRmPS groups (Supplementary Figure S3). The bacterial abundance in the AuR group did not change significantly in comparison with the Con group. These results indicated that the composition of dominant species in microbiota changed significantly due to the co-exposure of AuR and PS, especially due to co-exposure with nanoscale PS.

**Figure 6:**
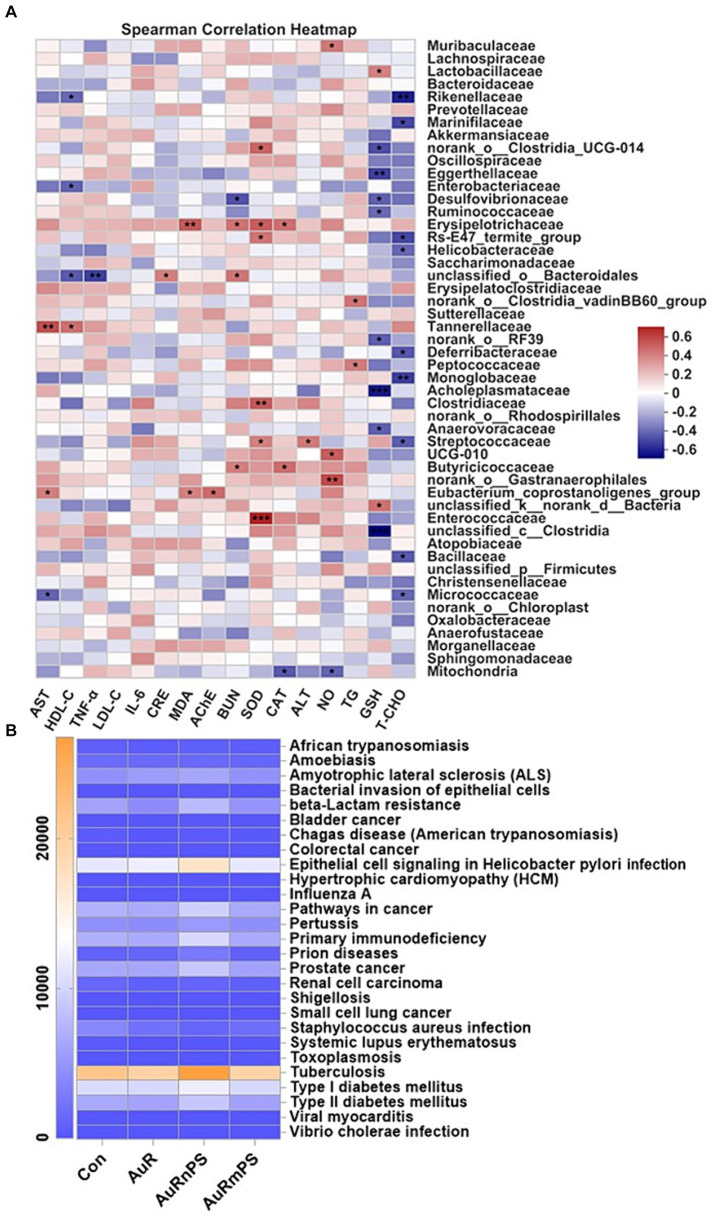
**(A)** Correlations between biomarkers and relative abundance of gut microbiota at the family level. **(B)** Heatmap of gut microbial genes involved in human diseases at the Level 3 of the KEGG pathway annotation. *p < 0.05, **p < 0.01, **p < 0.001.

Correlations between biochemical biomarkers and microbial composition were performed (Figure 6A). Gut microbiota significantly correlated with biomarkers of hepatotoxicity (AST, ALT, SOD, CAT, GSH, and MDA), biomarkers of nephrotoxicity (CRE and BUN), biomarkers of neurotoxicity (AChE and NO), inflammatory cytokines (TNF-α), and lipid metabolism (TG, TCHO, and HDL-C). There was a selective vulnerability in biochemical markers against the gut microbiota and a certain biomarker was generally significantly affected by a few specific microbiomes. For example, alterations in liver biomarkers were associated with *Lactobacillaceae*, *Eggerthellaceae*, *Desulfovibrionaceae*, *Ruminococcaceae*, *Erysipelotrichaceae*, *Tannerellaceae*, *Acholeplasmataceae*, *Clostridiaceae*, *Anaerovoracaceae*, *Streptococcaceae*, *Butyricicoccaceae*, *Enterococcaceae*, *Clostridia*, *Micrococcaceae*, and *Mitochondria* etc., alterations in kidney biomarkers were associated with *Desulfovibrionaceae*, *Erysipelotrichaceae*, *Bacteroidales*, and *Butyricicoccaceae*; alterations in neuron biomarkers were associated with *Muribaculaceae*, *Gastranaerophilales*, and *Mitochondria* etc., inflammation response was associated with *Bacteroidales*; lipid metabolism was associated with *Rikenellaceae*, *Marinifilaceae*, *Enterobacteriaceae*, *Helicobacteraceae*, *Bacteroidales*, *Tannerellaceae*, *Deferribacteraceae*, *Peptococcaceae*, *Monoglobaceae*, *Streptococcaceae*, *Bacillaceae*, and *Micrococcaceae* etc.

By reference to the KEGG pathway analyses at the Level 3 on the genes of gut microbiota, we found that the AuRnPS group was associated with primary immunodeficiency, increased risks of developing cancers, prion diseases, and Type-II diabetes, and increased beta-Lactam resistance (Figure 6B; Supplementary Figure S4). Results of KEGG pathway analyses at the Level 1 and Level 2 confirmed the finding of increased health risks in the AuRnPS groups. Specifically, the AuRnPS group exhibited altered genetic abundances in cellular process, environmental information processing, genetic information processing, human disease, metabolism, and organismal systems at the Level 1 KEGG analyses (Supplementary Figure S5). At the KEGG Level 2 analyses, the AuRnPS group showed higher genetic abundances in gut microbiota related to cancers, immune system diseases, infectious diseases, metabolic diseases, and neurodegenerative diseases (Supplementary Figure S6). These results consistently suggested the increased health risks in the AuRnPS exposure. By contrast, the AuRmPS group showed negligible alterations in the genetic abundances in comparison with the AuR group, suggesting that the microbial alterations depend on the size of co-exposed plastics particles.

## 4 Discussion

In current study, we aimed at revealing the multifaceted aspects of toxicology induced by the co-exposure of AuNP and PS, including hepatotoxicity, nephrotoxicity, and neurotoxicity. Moreover, influences on inflammation, lipid metabolism, and gut microbiota were systematically investigated. Related findings will facilitate the precision design of AuNPs-based therapies. For example, the enhanced hepatotoxicity induced by co-exposure suggests the extraordinary necessity of controlling co-exposure with plastics products for patients with liver diseases (e.g., hepatic carcinoma). Larger alteration in gut microbiota induced by AuRnPS suggests that preventing co-exposure with nanoscale PS is of higher priority when caring patients with gut-related diseases.

**Figure 7:**
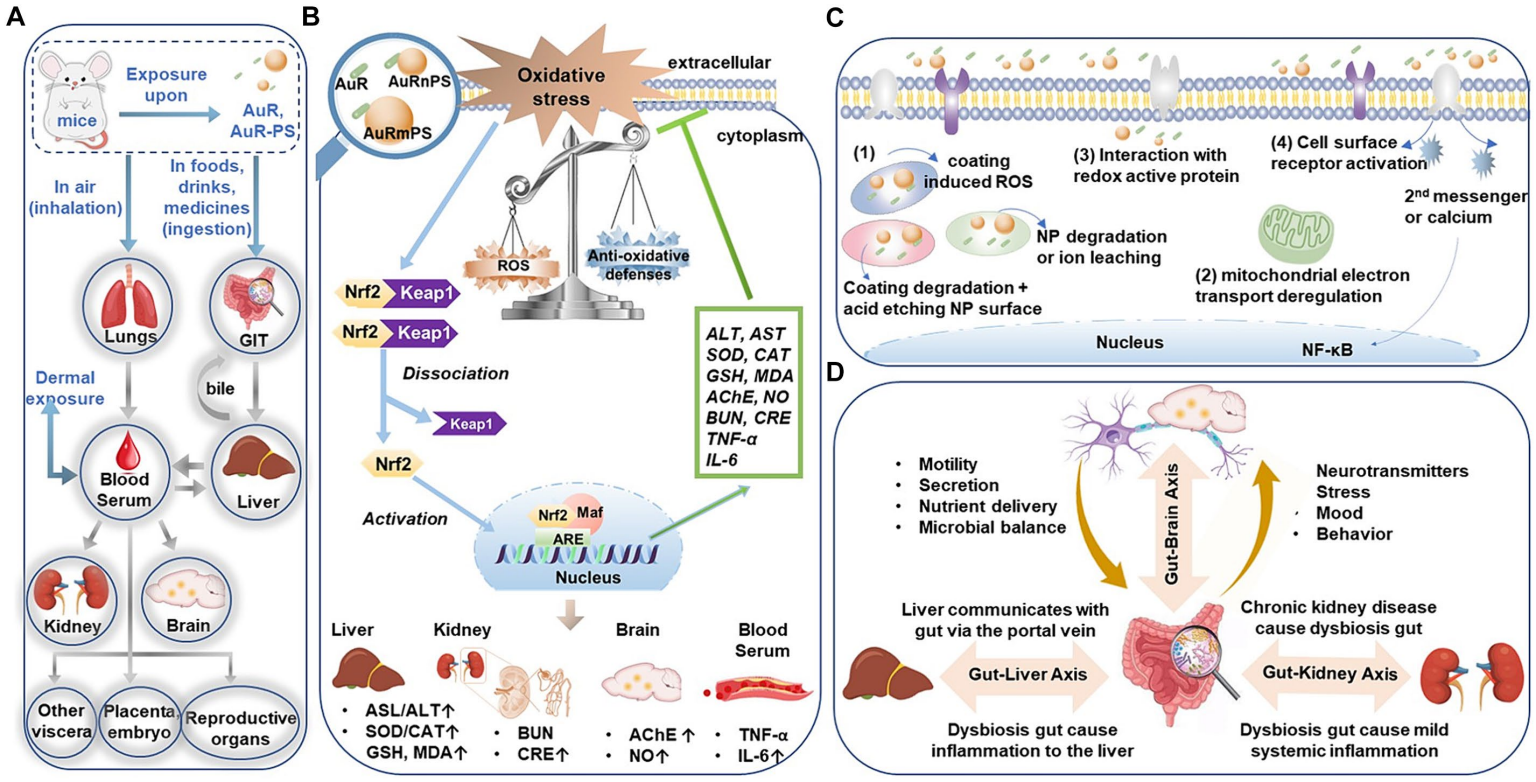
**(A)** Entry and translocation pathways of micro−/nano- particles. **(B)** Schematic illustration of the ROS-activated Keap1-Nrf2/ARE signaling pathway induced by the co-exposure of AuR with PS micro−/nanoplastics. **(C)** Different pathways for nanoparticles to generate ROS. **(D)** Bidirectional or multidirectional communication links (axes) between the gut microbiota and liver, kidney, or brain, respectively. GIT, gastrointestinal tract. ROS, Reactive oxygen species. Keap 1, Kelch-like ECH-associated protein 1. Nrf2, nuclear factor etythroid-2 related factor 2. ARE, antioxidant response element. NP, nanoparticles. NF-kB, nuclear factor-kappa B.

Plastic contamination is a rapidly growing risk factor for public health. Apart from the daily exposure or uptake (via diet), patients are associated with plastic exposure when receiving treatments or using medical device. Distribution of these plastic particles in tissues and organs, translocation across cellular membrane, and accumulation in the cellular compartment are the typical process, as outlined in Figure 7A, for plastic particles to induce toxicity via oxidative stress and inflammatory responses (Chang et al., 2020). With the rapid developments of nanobiotechnologies (Yuan et al., 2019) and nanoparticle-based therapies, systemic toxicological investigations (Gunsolus and Haynes, 2016) were becoming more and more critical in accurately assessing the therapeutic risks and potential iatrogenic side effects.

**Hepatotoxicity**. Hepatic injuries following co-exposure (AuR and PS) may most likely be driven by the oxidative stress, as supported by the increased SOD activity, CAT activity, and MDA level. Increased SOD and CAT activities can be attributed to Nrf2 (nuclear factor etythroid-2 related factor 2; Figure 7B), which is a component of Nrf2/ARE (antioxidant response element) signaling pathway orchestrating the transcription of the antioxidant genes of SOD and CAT and promoting the related protein expression at the early stage of oxidative stress (Jaiswal, 2004). The significant positive correlation between the stress-responsive enzyme activity (i.e., SOD or CAT) and lipid peroxidation product (MDA) level (Supplementary Figure S7) suggests increased substrate and activity of antioxidant enzymes following excessive ROS. Nanoparticles can generate ROS in different pathways (Figure 7C): (1) direct reactivity on the surface of metal nanoparticle; degradation of the surface coating (e.g., CTAB) or of the whole nanoparticle with the consequent release of free ions; (2) interactions with cellular organelles such as mitochondria following the alteration of electron transport chains; (3) interaction with redox active protein; (4) interaction with cell surface receptors and activation of intracellular signaling pathways (Soenen et al., 2011; Caballero-Díaz and Valcárcel, 2014). For instance, AuNPs generate ROS from dioxygen due to their high surface/volume ratio and the specific electronic configuration of the surface Au atoms (Pan et al., 2009). Moreover, the oxidative stress can be caused by secondary effects derived from nanoparticles endocytosis and interaction with intracellular biomolecules or organelles. Oxidative stress is associated with protein and polyunsaturated fatty acid oxidation, which ultimately leads to a profound alteration in mitochondrial function and thereafter cell death. The liver cellular uptake for positively charged nanorod were warranted by electrostatic interactions between the surface of AuR and the cell membrane. While the negatively charged PS surface is likely to adsorb more positively charged AuR (Supplementary Figure S8), so it is likely to induce severer uptake and accumulation in the organs. These findings are consistent with the literature report that microplastics aggravated the toxicity of organophosphorus flame retardants (Deng et al., 2018).

**Nephrotoxicity**. Our finding of enhanced nephrotoxicity after co-exposure is consistent with the previous study surrogating acute exposure to titanium dioxide nanoparticles (Wang J. et al., 2007). Nanoparticles can interact with different parts of the renal clearance system after glomerular filtration to initialize renal damage (Makhdoumi et al., 2020). In addition, ROS-mediated cytotoxicity has also been considered as a major mechanism of nephrotoxicity induced by metal-based nanoparticle exposure (Figures 7B,C; Wang et al., 2017).

**Neurotoxicity**. AChE activity in brain is a commonly used biomarker for neurotoxicity (Durieux et al., 2011; Guilhermino et al., 2018). The increase AChE activity will possibly lead to a reduction of cholinergic neurotransmission efficiency (Ferreira et al., 2012). Increased NO content in arteries indicates hyperperfusion to supply the increased metabolic requirement in impaired neuron cells with reduced efficiency. Excessive NO is neurotoxic and can induce apoptosis of various nerve cells, constituting a pathogenic risk factor to neurodegenerative diseases such as Alzheimer’s disease. In literature (Calderón-Garcidueñas et al., 2003), there are three possible ways that nanoparticles can cause central nervous system damage: (a) nanoparticles entering the body trigger tissue inflammatory response. For example, inhaled nanoparticles are deposited in alveolar tissue and causes lung inflammation, allowing inflammatory cytokines to enter the blood circulation (Figure 7A), systemic inflammatory response arises to cause functional damages; (b) nanoparticles transport into the central nervous system to activate microglia, leading to the large expression of neurotoxic molecules (e.g., free radicals and inflammatory factors) and resulting in nerve damage (Figure 7B); (c) nanoparticles impair the normal function of neurons to induce toxicity to the limbic system during transportation along the sensory nerves.

**Inflammation**. Metal nanomaterials entering the immune system can induce and accelerate inflammatory responses by stimulating immune cells to produce pro-inflammatory factors and chemokines (Zhou et al., 2012). Microplastics exposure can increase the levels of pro-inflammatory cytokines in mice (Li et al., 2020). Meanwhile, ROS oxidizes lipids and proteins, leading to ultimate cell and tissue damage to promote inflammation (Figures 7B,C; Bauernfeind et al., 2011). Our observations of enhanced inflammatory responses in AuR, AuRnPS, and AuRmPS mice were consistent with those reports.

**Gut microbiota**. Our results showed that the diversity and composition of the gut microbiota in mice were changed significantly after exposure to AuR, AuRnPS, and AuRmPS. The PCoA figure shows a general homogeneous clustering between Con, AuR, and AuRmPS, with higher variability for AuRnPS at the family level. This is reflected in the Phylum taxa bar plot, which shows a higher abundance of Firmicutes in AuRnPS. Our finding supports the notion that the alteration in gut microbiota is dependent on the size of co-exposed PS with AuR. Different microbiota is involved in different pathogenic process or physiological process. For example, Bacteroides play a variety of important metabolic functions: participation in the oxidation of long-chain fatty acids, utilize polysaccharides, the regulation of acyl enzyme A levels, and affecting protein and stability by changing the degree of protein acetylation (Hooper et al., 2002; Ma et al., 2021). Increased Firmicutes in the cecum promotes nutrient absorption and correlates with the development of obesity (Ley et al., 2005). *Actinobacteria* are one the four major phyla of the gut microbiota, although they represent only a small percentage, are pivotal in the maintenance of gut homeostasis (Binda et al., 2018). *Actinobacteria* include three main anaerobe families (*Bifidobacteria*, *Propionibacteria* and *Corynebacteria*) and an aerobe family (*Streptomyces*). The most represented in the human gut are *Bifidobacteria*, which can modulate immune-inflammatory and autoimmune response by inducing T-Cell (Lyons et al., 2010; Binda et al., 2018). In addition, *Actinobacteria* are associated with the biodegradation of resistant starch (Ryan et al., 2006). The *Eggerthellaceae* shown in this study is a family within the order *Coriobacteriales* (Phylum *Actinobacteria*), which can metabolize fiber to lactate and acetate (Clavel et al., 2014).

Due to the smaller size, nanoscale PS holds higher permeability across the intestinal barrier to enter the systemic circulatory system than microscale PS. Thereafter, excretion process of nanoscale PS from the body is slower to introduce long-lasting damage. In comparison with the single exposure of AuR, the co-exposure with PS changed the distribution pattern and accumulation of AuR in tissues. PS can interact with microorganisms and have the potential to serve as a unique microbial habitat (McCormick et al., 2014). The small size of nanoscale PS may contribute to a higher affinity to intestinal villus, thereby allowing more accumulation of nanoparticles in the intestinal system to induce a larger alteration in the gut microbiota. The absorption rate of plastics particles by the digestive tract is directly related to the subsequent biological effects induced. Different *in vitro* gut models were utilized to measure the absorption rate of PS (50–500 nm) with values ranging from 1.5 to 10% due to the differences in particle size and surface properties (Walczak et al., 2015). The absorption rate of PS in the intestinal tract decreases with the particle size, e.g., the absorption rate of 2 μm PS in the intestinal tract is 0.04–0.3% (Carr et al., 2012). Moreover, the human colonic mucosal tissue model showed that membrane transport efficiency of 3 μm microparticles is lower than that of 250 nm nanoparticles (Schmidt et al., 2013). An additional factor to promote the particle absorption may be the impaired tissue permeability induced by the inflammatory responses (Schmidt et al., 2013).

**Correlations between biomarkers and gut microbiota**. Both liver and gastrointestinal tract (GIT) are components in the digestive system and play important roles in the metabolic synthesis, detoxification process, and immune responses. The interaction of the gut-liver axis (Albillos et al., 2020) is established by the vascular access of the portal vein for direct delivery of gut-derived products to the liver, and the feedback of bile and antibodies from the liver to gut (Figures 7A,D). In this study, AuR and PS with different size disrupted the intestinal mucosa, resulting in increased intestinal permeability and inflammatory infiltration. At the family level, 20 of the top 50 abundant species were significantly associated with markers of liver injury (i.e., AST, ALT, SOD, CAT, GSH, and MDA; Figure 6B). In addition, many studies (Leclercq et al., 2014; Inoue et al., 2018; Porras et al., 2019) have reported that gut microbiota affects the occurrence and development of liver-related diseases through the gut-liver axis, and microbial factors are the driving force for many different stages of liver diseases, such as hepatic steatosis, liver inflammation, fibrosis development, and tumorigenesis. Our current study and these existing reports consistently suggest that the gut microbiota is associated with the pathogenesis of various diseases. The exact underlying mechanism at the molecular level requires further investigations.

Many studies have found that intestinal microecological disturbances are closely related to the abnormal immune function of patients with chronic kidney disease and are potential causes of persistent immune deficiency and systemic inflammation in patients with chronic kidney disease. The concept of gut-kidney axis has been proposed to explain the interplay between gut and kidney with a two-way regulation process (Meijers and Evenepoel, 2011; Pahl and Vaziri, 2015). Moreover, the interplay among the gut, kidneys, and immunity was explored. It was found that patients with chronic kidney diseases will experience intestinal flora disturbances and thereby aggravate kidney damages through the bridge of immune response (Knauf et al., 2019). Tight junctions of the intestinal epithelia will be damaged to increase the intestinal permeability as a result of toxicological response. Toxins will pass through the intestinal wall with leaky permeability, enter the blood circulation to activate the monocyte–macrophage system, and cause profound releases of cytotoxic substances to aggravate the systemic micro-inflammatory response and endotoxemia to peril the renal functions (Figure 7D). In this study, at the family level, four gut microbiotas were significantly correlated with markers of kidney damages (Figure 6B).

Gut microbiota can also affect the brain function and behavior through the gut-brain axis (Teng et al., 2022), as evidenced by the correlation between AChE activities and microbiota, and between NO level and microbiota (Figures 6B, 7D). Human gut microbiomes affect human brain in following manners: (1) Bacterium signals the innate immune system with low-grade tonic stimulation (for example, lipopolysaccharides). Hyperstimulation caused by bacterial dysbiosis, bacterial overgrowth in small intestine, or increased intestinal permeability may produce systemic and/or central nervous system inflammation; (2) Bacterial proteins cross-react with human antigens and induce dysfunctional responses in the adaptive immune system; (3) Bacterial enzymes can produce neurotoxic metabolites, including D-lactic acid, ammonia, and short-chain fatty acids; (4) Gut microbes can produce the same hormones and neurotransmitters as human. Bacterial receptors for these hormones influence microbial growth and virulence; (5) Gut bacteria directly stimulate afferent neurons of the enteric nervous system, sending signals to the brain through the nerve. Through these different mechanisms, gut microbes help shaping the structure of sleep and the stress response of the hypothalamic–pituitary–adrenal axis. The gut-brain axis affects memory, mood, and cognition, and are clinically associated with various disorders, including alcoholism, chronic fatigue syndrome, fibromyalgia, and restless legs syndrome. In current study, we found five taxa associated with significant alterations in biomarkers for neurotoxicity (Figure 6B).

These basic and systematic toxicological data provide the basis for the selective vulnerability of different organs to AuNPs exposure and its co-exposure with micro−/nano-plastics. The findings could help explore ways to reduce toxicity and thus improve the biosafety of AuNP and microplastics. At present, the biological effects and toxicological mechanisms of nanomaterials and micro−/nano-plastics such as bioabsorption and transport are not comprehensive enough. Therefore, it is necessary to obtain basic toxicological data through toxicity experimental studies. It provides a reference and comparison for the establishment and regular updating of the background values of clinical tests of laboratory animals and provides basic information for the determination of environmental reference values for microplastics and the formulation of standards. In addition, endpoint biochemical markers were measured in this study. *In vivo* non-invasive functional MRI can be performed in future studies to track potential perturbations in macroscopic organ function, e.g., blood–brain barrier, oxygen metabolism, cerebral blood flow (Wei et al., 2020; Liu et al., 2023).

## 5 Conclusion

In comparison with the single exposure of AuR, co-exposure of AuR and PS exacerbates the hepatotoxicity, nephrotoxicity, neurotoxicity, and inflammation. Meanwhile, the alterations in the diversity/distribution of gut microbiota and the relative abundance of bacteria are more prominent under the co-exposure of AuR and nano-size PS. These basic toxicological data obtained through systematic chemical and biological assessments provide the underpinnings for the selective vulnerability of different organs toward the exposure of AuNP and its co-exposure with microplastics, facilitate the exploration of methods to reduce toxicity, and thereafter improve the biosafety of using AuNP and microplastics. Based on our finding, plastics exposure controlling is recommended in AuNPs-based therapies and prevention against nano-size plastics is of higher priority.

## Data Availability

The original contributions presented in the study are publicly available. This data can be found here: https://doi.org/10.6084/m9.figshare.26828863.v1.

## Footnotes

^1^http://www.genome.jp/kegg/

^2^http://huttenhower.sph.harvard.edu/galaxy

^3^https://cloud.majorbio.com
