# Supplementary material for: Toxicity of gold nanoparticles complicated by the co-existence multiscale plastics
Source: Front Microbiol. 2024 Aug 29;15:1447046. doi: 10.3389/fmicb.2024.1447046 (PMC11392435; doi:10.3389/fmicb.2024.1447046)
Supplement: Supplementary file 1 [file Table_1.DOCX]

Supplementary Material

**Toxicity of gold nanoparticles complicated by the co-existence multiscale plastics**

Lan Zhang^1,*^, Yuyang Ma^1,2^, Zhiliang Wei^3^, Luyang Wang^1^

^1^ *College of Food Science and Engineering, Ocean University of China, Qingdao 266003, China*

^2^ *School of Pharmacy, Binzhou Medical University, Yantai 264003, China*

^3^*Department of Radiology & Radiological Science, Johns Hopkins University School of Medicine, Maryland, USA*

^*^E-mail: lanzhang_ouc@outlook.com


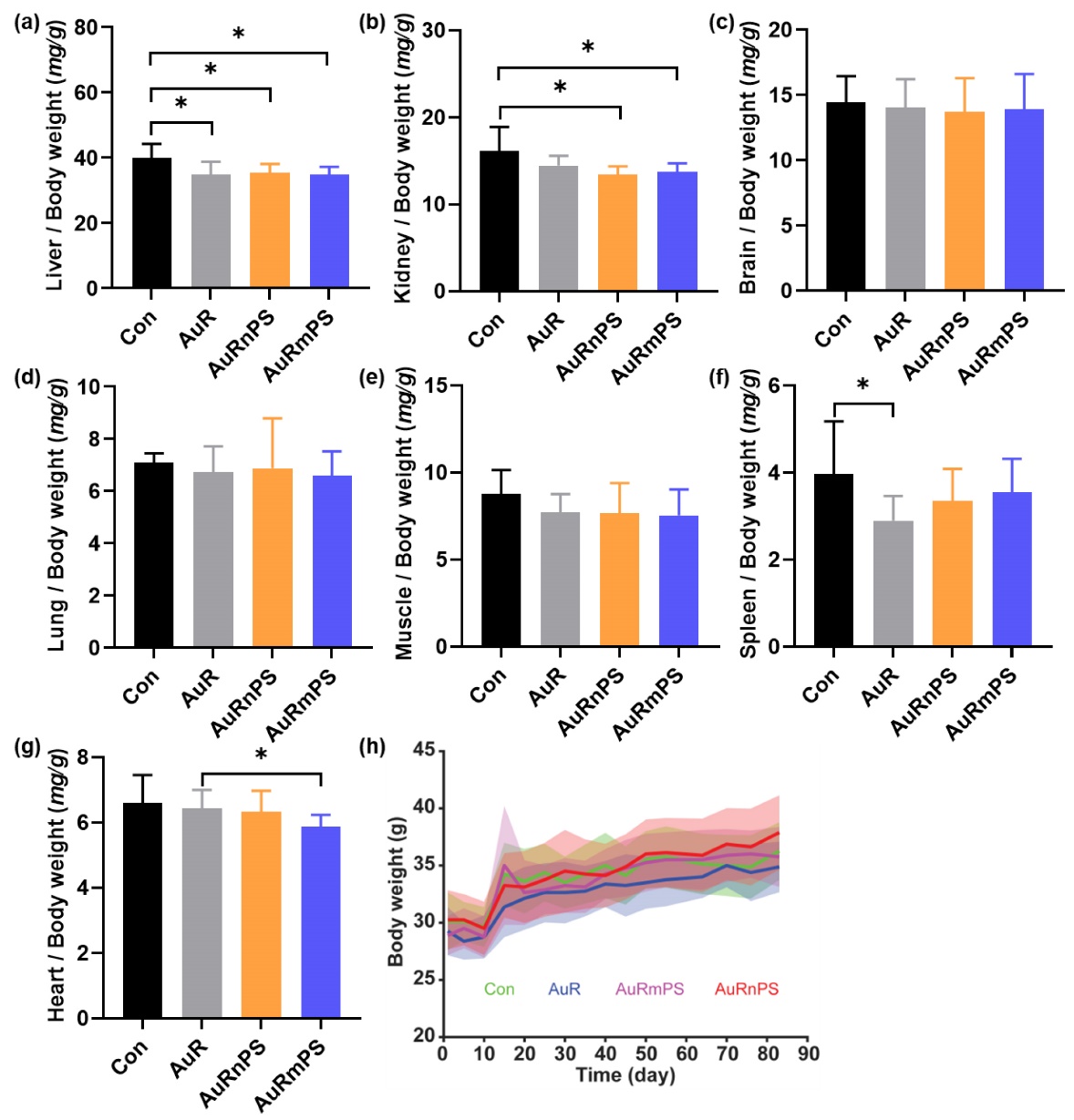


**Figure S1.** Effects of AuR and AuR-PS on organ coefficients (a-g) and body weight (h). (a) shows the organ coefficient comparisons among four groups for liver; (b) for kidney; (c) for brain; (d) for lung; (e) for muscle; (f) for spleen; (g) for heart; and (h) for changes of body weight during the 90 consecutive days under exposure. * denotes *P*<0.05.


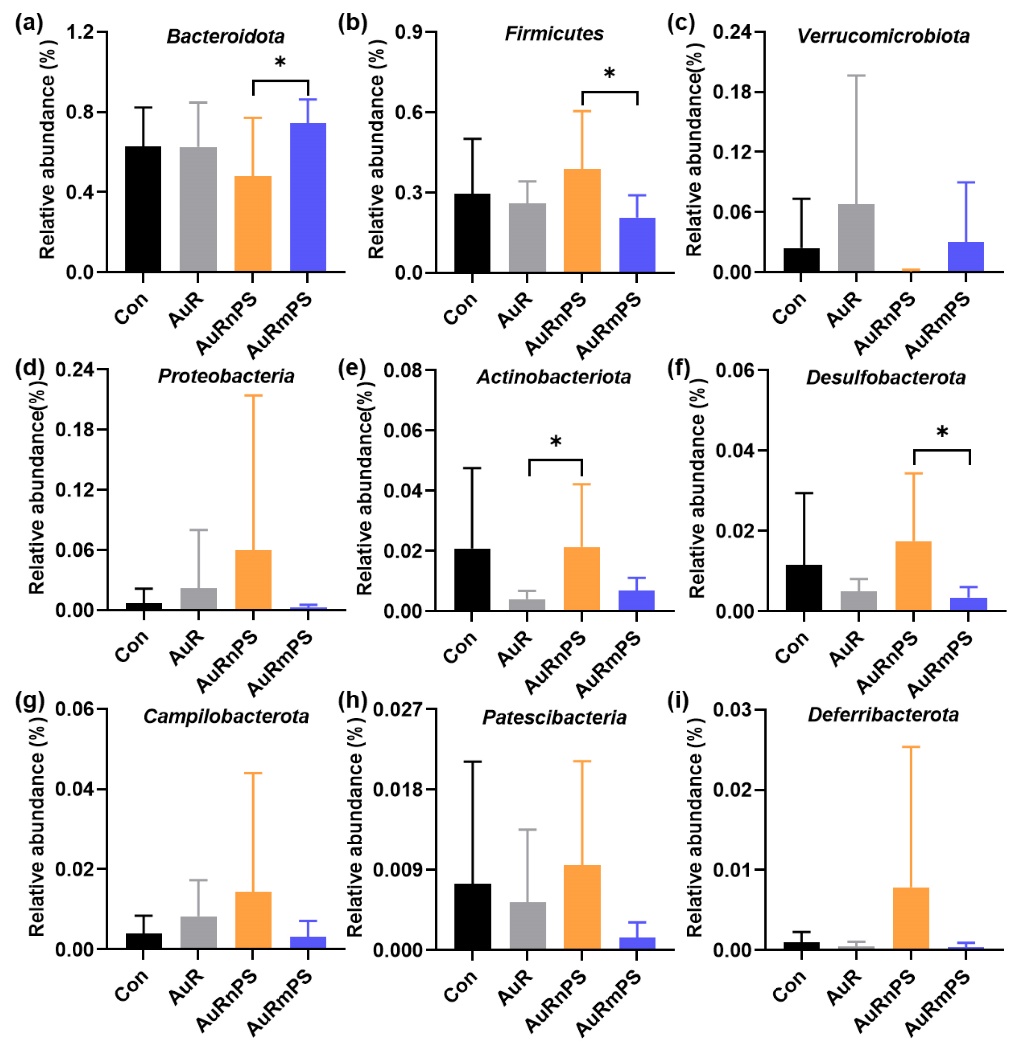


**Figure S2**. Effects of AuR and co-exposures with PS (500 nm, 5 μm) on relative abundance of species in fecal microbial composition oat the phylum level. (a) shows the abundance comparisons among four groups for *Bacteroidota*; (b) for *Firmicutes*; (c) for *Verrucomicrobiota*; (d) for *Proteobacteria*; (e) for *Desulfobacterota*; (f) for *Actinobacteriota*; (g) for *Campilobacterota*; (h) for *Patescibacteria*; and (i) for *Deferribacterota*. *Denotes *P*<0.05.


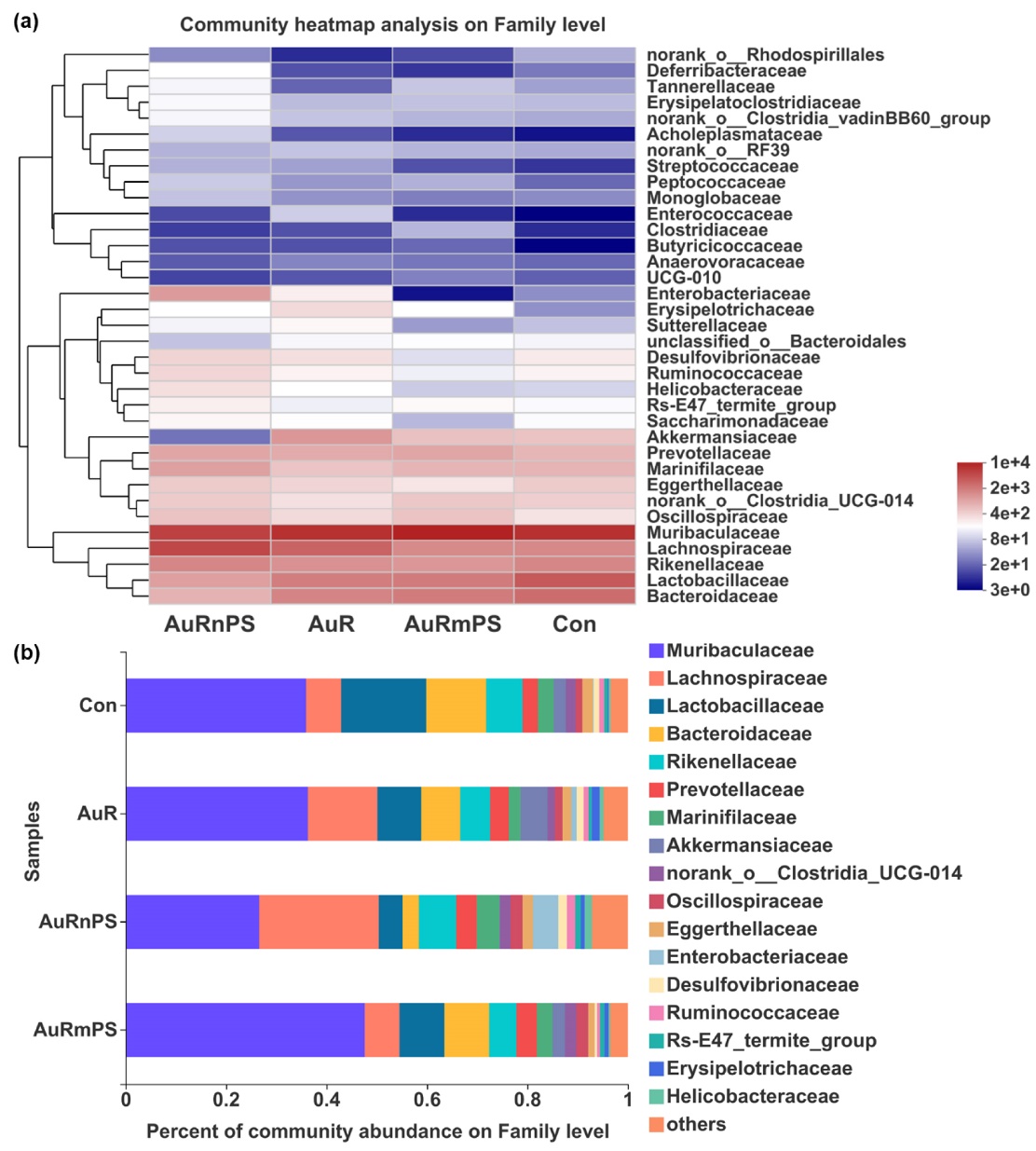


**Figure S3**. Effects of AuR and co-exposures with PS (500 nm, 5 μm) on heatmap of the relative abundance of gut microbiota at the family level (a) on compositional changes of gut microbiota at the family level (b).


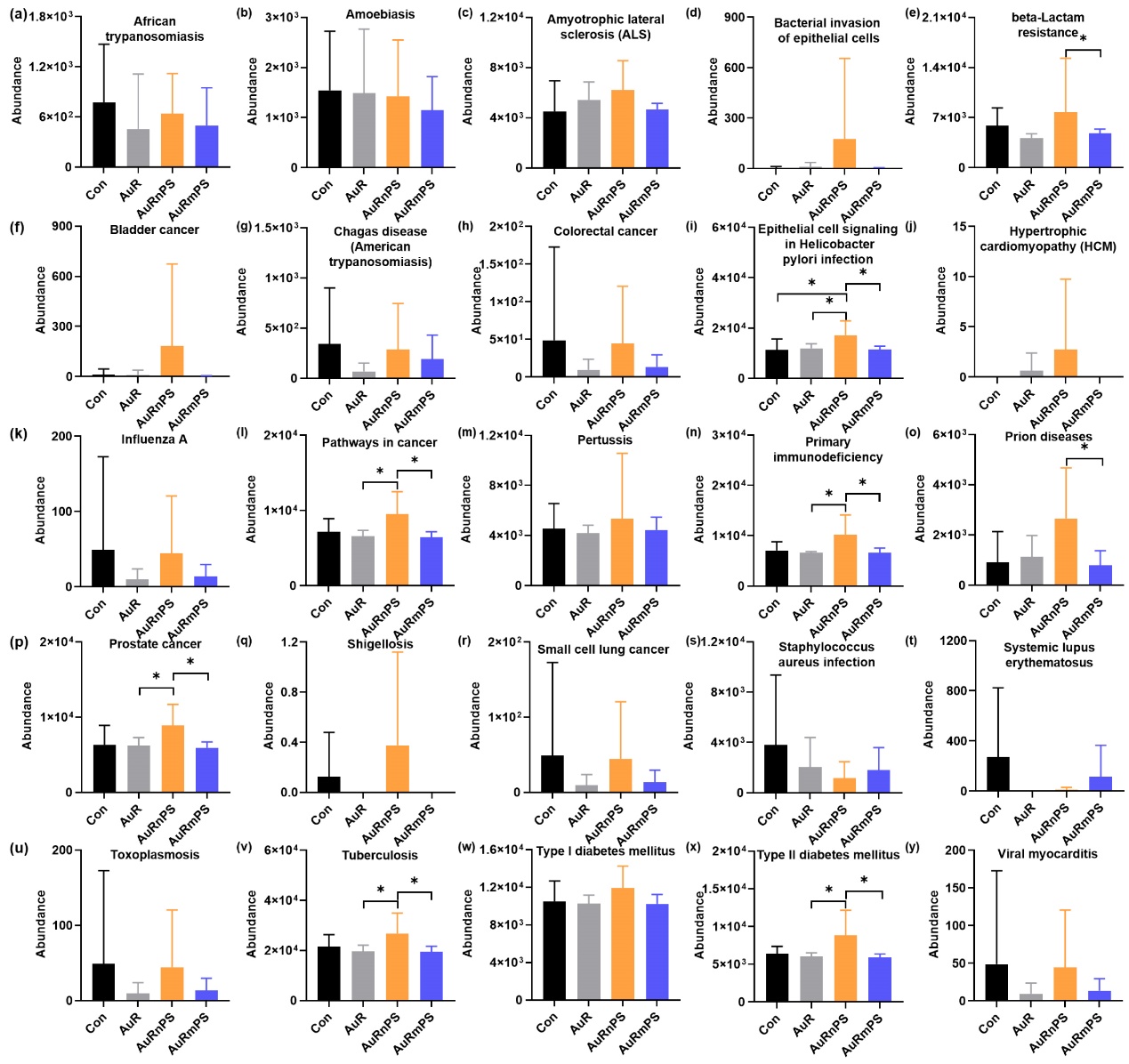


**Figure S4**. Gut microbial genes involved in human diseases at the Level 3 of KEGG pathway annotation. (a) shows the genetic abundance for African trypanosomiasis; (b) for amoebiasis; (c) for amyotrophic lateral sclerosis; (d) for bacterial invasion of epithelial cells; (e) for beta-Lactam resistance; (f) for bladder cancer; (g) for chagas disease (American trypanosomiasis); (h) for colorectal cancer; (i) for epithelial cell signaling in helicobacter pylori infection; (j) for hypertrophic cardiomyopathy; (k) for influenza A; (l) for pathways in cancer; (m) for pertussis; (n) for primary immunodeficiency; (o) for prion diseases; (p) prostate cancer ; (q) shigellosis; (r) small cell lung cancer; (s) staphylococcus aureus infection; (t) systemic lupus erythematosus; (u) toxoplasmosis; (v) tuberculosis; (w) type I diabetes mellitus; (x) type II diabetes mellitus; and (y) viral myocarditis. *denotes *P*<0.05.


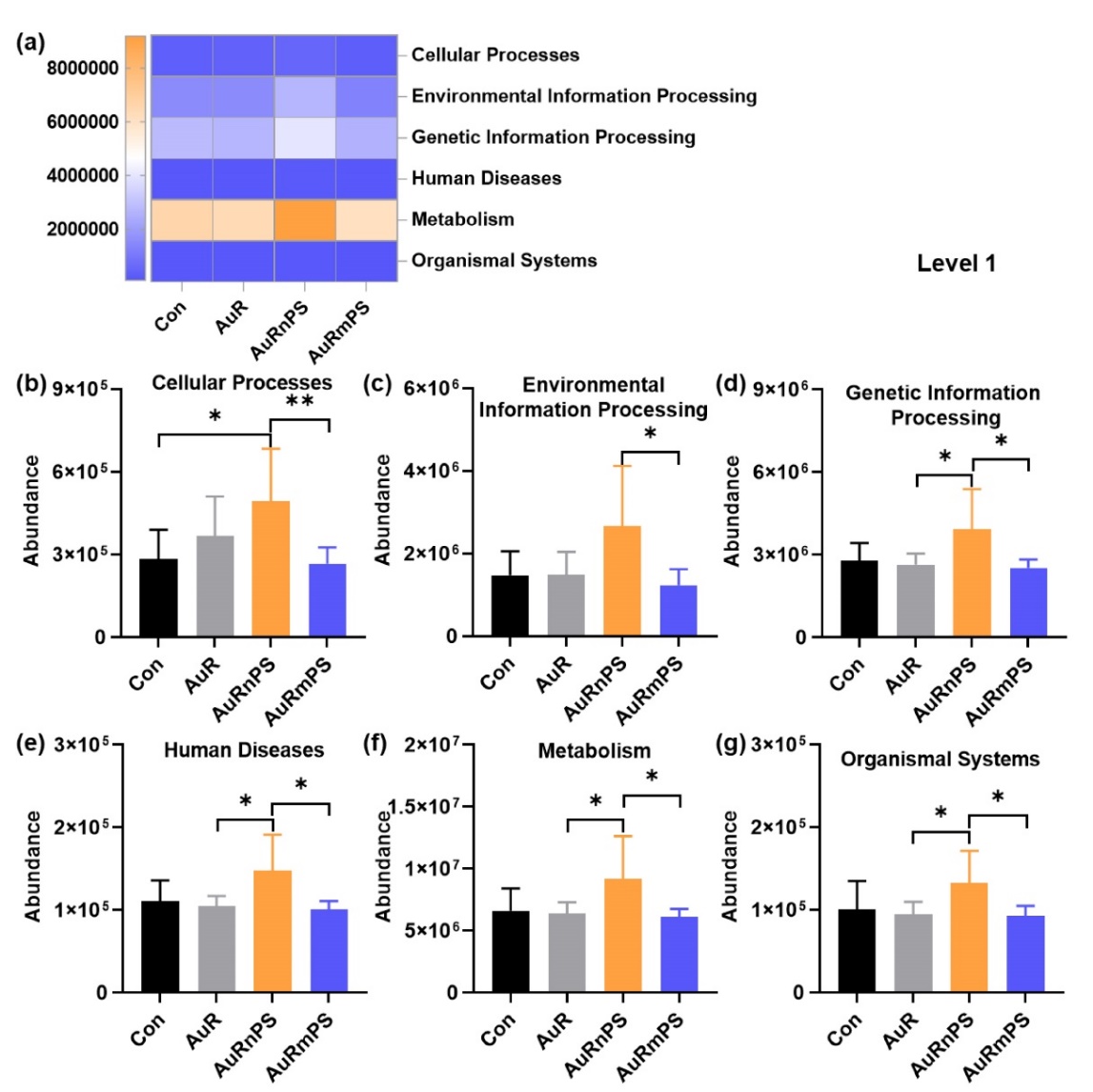


**Figure S5**. (a) Heatmap of gut microbial genes at the Level 1 of KEGG pathway annotation. (b) shows the genetic abundance for cellular processes; (c) for environmental information processing; (d) for genetic information processing; (e) for human diseases; (f) for metabolism; and (g) for organismal systems. *denotes *P*<0.05, ***P*<0.01.


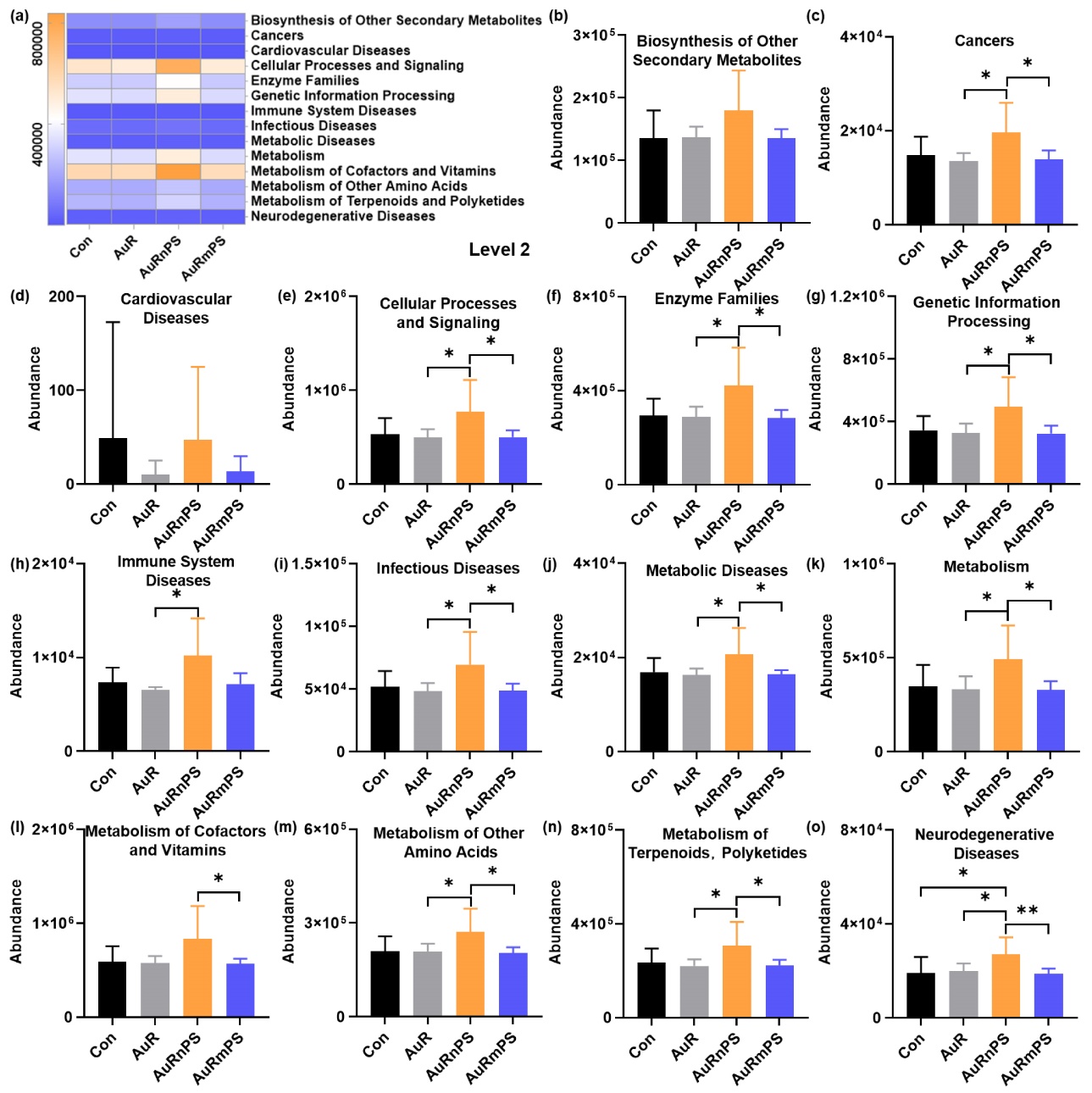


**Figure S6**. (a) Heatmap of gut microbial genes at the Level 2 of KEGG pathway annotation. (b) shows the genetic abundance for biosynthesis of other secondary metabolites; (c) for cancers; (d) for cardiovascular diseases; (e) for cellular processes and signaling; (f) for enzyme families; (g) for genetic information processing; (h) for immune system diseases; (i) for infectious diseases; (j) for metabolic diseases; (k) for metabolism; (l) for metabolism of cofactors and vitamins; (m) for metabolism of other amino acids; (n) for metabolism of terpenoids and polyketides; and (o) for neurodegenerative diseases. *denotes *P*<0.05, ***P*<0.01.


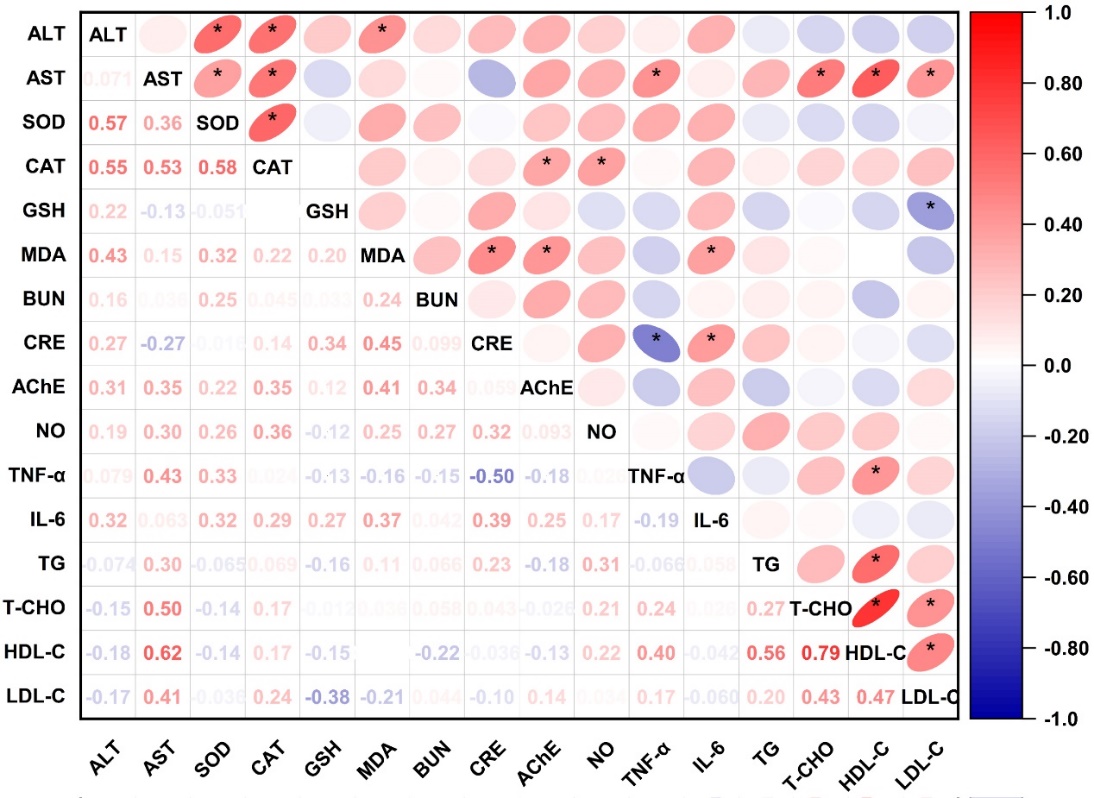


**Figure S7**. Correlations between different biochemical measurements. * denotes *P*<0.05.


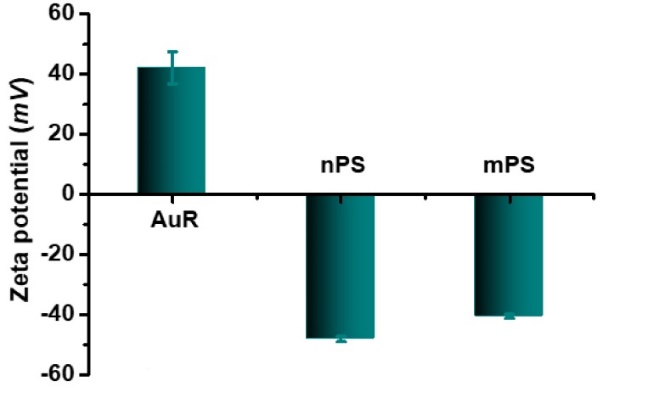


**Figure S8**. Zeta-potential (mV) of AuR, nPS, and mPS in water.
